# Using the lagging strand to study chromosome replication

**DOI:** 10.1186/1756-8935-6-S1-O2

**Published:** 2013-03-18

**Authors:** S McGuffee, D Smith, I Whitehouse

**Affiliations:** 1 Molecular Biology Program, Memorial Sloan-Kettering Cancer Center, 1275 York Avenue, New York, New York 10065, USA

## 

DNA replication is inherently asymmetric. Synthesis of the lagging strand occurs discontinuously and necessitates the repeated production of Okazaki fragments. Recently, using transient DNA ligase I inactivation, we have been able to capture and analyze short DNA molecules produced on the lagging strand. These Okazaki fragments are typically less than 500bp in length and are generally sized according to the nucleosome repeat. Using deep sequencing, we have found that Okazaki fragment synthesis is strongly influenced by nucleosomes as well as certain other DNA bound factors. Our methodology also provides a genome-wide view of DNA replication: allowing the first detailed measurements of the efficiencies of all replication origins and regions of termination. We provide evidence that S-phase follows a distinct temporal program dominated by replication origins firing with high probability within distinct time intervals. Contrary to expectation, we find that centromeres and highly transcribed regions are not strong determinants of replication termination; rather, termination generally occurs midway between two adjacent replication origins at positions dictated by the relative firing times of those origins. Sites of termination are, therefore, indicative of origin firing time – allowing us to faithfully reconstruct the temporal dynamics of the replication program using data from a single asynchronous culture.

